# A cross-sectional study of non-suicidal self-injury in a Chinese adolescent inpatient cohort

**DOI:** 10.3389/fpsyt.2023.1109334

**Published:** 2023-05-24

**Authors:** Ke Sun, Anni Li, Yichen Li, Jun Xie, Yonghao Tong, Jun Ma, Yong Wu

**Affiliations:** ^1^Department of Child and Adolescent Psychiatry, Wuhan Mental Health Center, Wuhan, Hubei, China; ^2^Affiliated Wuhan Mental Health Center, Tongji Medical College, Huazhong University of Science and Technology, Wuhan, Hubei, China; ^3^Research Center for Mental Health and Neuroscience, Wuhan Mental Health Center, Wuhan, Hubei, China

**Keywords:** non-suicidal self-injury, inpatient, adolescent, depression, gender

## Abstract

**Background:**

Non-suicidal self-injury (NSSI) is a significant predictor of completed suicide and is increasingly recognized as a serious public health concern. Multiple factors, including social, familial, mental, and genetic factors could influence the occurrence of this behavior. Identifying the early risk factors is important for screening and preventing this behavior.

**Methods:**

Here, we recruited a total of 742 adolescent inpatient participants from a mental health center and conducted a series of diagnostic interviews and questionnaires to assess NSSI behavior and other events. Bivariate analysis was used to detect differences between groups in NSSI and non-NSSI. Then, binary logistic regression was fitted to identify predictors of NSSI as a function of these questionnaire scores.

**Results:**

Of the 742 adolescents examined, a total of 382 (51.5%) participants engaged in NSSI. Bivariate analysis showed that age, gender, depression, anxiety, insomnia, and childhood trauma was significantly associated with NSSI. Logistic regression results suggested that females had 2.43 higher odds of engaging in NSSI when compared to their male counterparts (OR = 3.43, 95%CI = 2.09–5.74, *p* = 1.70 × 10^−6^). Depression was a primary risk predictor for NSSI with each additional increase in symptoms of depression increasing the odds of engaging in NSSI by 18% (OR = 1.18, 95%CI = 1.12–1.25, *p* = 2.25 × 10^−8^).

**Conclusion:**

More than half of the adolescent inpatients with psychiatric disorders have NSSI experience. Depression and gender were the risk factors for NSSI. Age at a specific range had a high prevalence of NSSI.

## Introduction

Non-suicidal self-injury (NSSI) is defined as the direct and deliberate destruction of one’s own bodily tissue, in the absence of any suicidal intent. This behavior typically involved cutting or carving the skin and has a consistent presentation cross-nationally (1). According to a 30 years epidemiology investigation study estimated in 2019, the aggregate lifetime prevalence of NSSI was 22.1% in children and adolescents (2). In particular, in inpatient cohorts with psychiatric disorders, the one-year prevalence of NSSI was as high as 62.2% (3). Moreover, the prevalence of NSSI had increased substantially in recent years (4). It was one of the strongest predictors of completed suicide (5, 6) and more attention should be paid to NSSI individuals from family and society.

So far, many risk factors have been identified to be associated with NSSI. First, a number of investigations suggested that childhood maltreatment (including sexual abuse, physical abuse, physical neglect, and emotional abuse), particularly childhood emotional abuse, was associated with NSSI (7, 8). However, other studies also found a non-significant relationship between childhood emotional abuse and NSSI, and sexual abuse featured prominently in NSSI (9, 10). Second, gender could have an influence on NSSI behavior, in which several studies showed girls were more likely to engage in NSSI than their male counterparts (9, 11, 12), though other studies failed to find any significant association between gender and NSSI (13–15). Third, depression and mood disorders were known predictors of NSSI and this association was found in almost all NSSI risk factor studies (9, 16, 17). Additionally, although studied less, genetics was also reported to be associated with NSSI, and the single nucleotide polymorphism (SNP) heritability of NSSI was estimated to be 13% (18). Adolescents who carried a mutation in the serotonin transporter gene *SLC6A4* showed an elevated likelihood of engaging in NSSI when exposed to severe interpersonal stress (19). Other factors, like maternal criticism (20), school bullying, peer rejection (21), cigarette smoking (22, 23), etc. were also the risk factors for NSSI.

Despite the increasing number of studies on NSSI, some results were inconsistent and most of these studies were conducted on people of European ancestry. Due to the large amount of NSSI behavior among Chinese children and adolescents (13, 24, 25), there is a need for studies of risk factors for NSSI in Chinese populations. Therefore, the aim of the current study is to investigate the risk factors for NSSI in a Chinese cohort. We collected a total of 742 inpatients aged from 11 to 19 in a mental health hospital in China. The self-rating scales and face-to-face interviews, including Self-Rating Depression Scale (SDS), Childhood Trauma Questionnaire-Short Form (CTQ-SF), and the Life Event Scale (LES), etc., were used to measure the depression state, childhood maltreatment, and negative life events, and so on. Then, the chi-square test and one-way analysis of variance (ANOVA) were conducted to compare the categorical variables and continuous variables between different genders, suicidal attitude, depression degree, etc. among children and adolescents who engaged in NSSI versus children and adolescents who did not engage in NSSI. Furthermore, binary logistic regression was chosen to establish the relationship between early childhood events, depression state, and NSSI behavior.

## Methods

### Sample collection

All the samples used in this study were collected from the child and adolescent inpatient ward of Wuhan Mental Health Center from October 2018 to December 2019. The basic population information, including gender, age, and grade was recorded. Individuals with other organic diseases or a history of smoking and drinking were excluded from this study. A total of 742 Han Chinese adolescents aged 11–19 years old (Mean = 14.69; SD = 1.69) were analyzed in this study. All participants wrote the informed consent, and for those participants aged under 18 years old, written informed consent of all legal guardians was gathered. The study protocol has been accepted by the ethics committee of the Wuhan Mental Health Center, China.

### Scale description

#### NSSI behavior

The dependent variable in this study was a single question about NSSI; “Have you, in the recent past 12 months, had an episode of self-injury without any intention of committing suicide?” The participants were asked to answer yes or no (1 = No, 2 = Yes).

#### PHQ-9 depression scale

The Patient Health Questionnaire-9 (PHQ-9) depression scale, which is based on 9 core symptoms of depression diagnosis described in DSM-IV, was used to measure depression state (26). Generally, this scale has 9 questions about “Over the last 2 weeks, how often have you been bothered by any of the following problems,” such as “Little interest or pleasure in doing things,” “Feeling down, depressed, or hopeless.” Each question has four choices (0 = Not at all, 1 = Several days, 2 = More than half the days, 3 = Nearly every day). Participants need to answer all 9 questions and the total score of the 9 items is the final depression score. The higher score indicates the more severe depression.

#### Generalized anxiety disorder 7-item scale

The GAD-7 scale, which is based on 7 core symptoms of anxiety described in DSM-IV, was used to measure the anxiety state (27). This scale had 7 questions about “Over the last 2 weeks, how often have you been bothered by any of the following problems,” like “Feeling nervous, anxious, or on edge,” “Not being able to stop or control worrying.” Like PHQ-9, each question had four choices (0 = Not at all, 1 = Several days, 2 = More than half the days, 3 = Nearly every day). Participants need to answer all 7 questions and the total score of the 7 items is the final anxiety score. Higher scores represent more severe anxiety symptoms.

### Childhood trauma questionnaire-short form scale

The CTQ-SF scale was used to assess the childhood negative experiences. The CTQ-SF was a widely used self-report assessment that measures exposure to five types of trauma - physical, sexual, and emotional abuse, and physical and emotional neglect (28). The CTQ-SF consisted of 25 trauma evaluation items and three validity items. Each type of maltreatment was represented by five items, like when I was growing up “I thought that my parents wished I had never been born” for emotional abuse, “Someone tried to touch me in a sexual way or tried to make me touch them” for sexual abuse, to provide adequate reliability and content coverage, while the three-item Minimization/Denial validity scale, like “I had the perfect childhood,” was used to detect the underreporting of maltreatment (28). Each of the abuse and neglect subscales items was rated on a 5-point Likert scale (1 = Never true, 2 = Rarely true, 3 = Sometimes true, 4 = Often true, 5 = Very often true) and a special maltreatment score was the total score of 5 relative items. Higher scores indicate more severe childhood maltreatment.

### Perceived devaluation and discrimination scale

The PDD scale was one of the most widely-applied measurements, which consisted of 12 questions, like “People would accept someone who had mental illness as a friend” (question 1) and “People think that seeking psychiatric services is a mark of personal failure” (question 5), that measure individual’s perceptions of social attitudes toward mental illness on stigma and discrimination (29). Each question had a five-point scale answer which ranged from “strongly agree (score 1)” to “strongly disagree (score 5).” Half of the questions of the scale (questions 5, 6, 7, 9, 11, 12) need to be reverse scored. The sum score of the 12 items was the total score of the PDD scale. Higher scores indicate greater severity of the perceived devaluation and discrimination.

### Pittsburgh sleep quality index scale

The PSQI was one of the most widely used sleep questionnaires, which consisted of 19 self-rating questions and 5 observer-rating questions that assess a number of aspects of sleep quality (30). Respondents were asked to indicate how frequently they have experienced certain sleep difficulties over the past month and to rate their overall sleep quality. Scores for each question ranged from 0 to 3, with higher scores indicating more acute sleep disturbances. Details about the question setting, reliability, and validity of this questionnaire could be found in the original publication (30).

### Suicide attitude questionnaire scale

Suicidal ideation was defined as seriously thinking of killing oneself and was measured by the QSA scale (31). This self-report questionnaire consisted of 29 questions rating on 4 sub-scale (a 5-point Likert scale each, to rate suicide attitudes from 1 = “strongly agree” to 5 = “strongly disagree”): attitude to suicide behavior (questions 1, 7, 12, 17, 19, 22, 23, 26, 29), attitude to suicide (question 2, 3, 8, 9, 13, 14, 18, 20, 24, 25), attitude to family members of suicide (question 4, 6, 10, 15, 28), and attitude to euthanasia (question 5, 11, 16, 21, 27). Thirteen items (questions 1, 3, 7, 8, 10, 11, 12, 14, 15, 18, 20, 22, 28) need reverse scoring. The sum score of a sub-scale is the total score with a higher score indicating less tolerance to suicide. A score less than 2.5 (code as 1) indicated an approbatory attitude to suicide; a score of 2.5–3.5 (code as 2) indicated a neutral attitude; scores more than 3.5 (code as 3) indicated an opposing attitude.

### Adolescent self-rating life events check list

The ASLEC was used to assess whether the negative events occurred, and if any, how serious the impact is on the participant in the past year (32). It listed 26 negative life events, like “beaten by parents” and “failure in an exam,” covering 5 aspects: interpersonal stress, study pressure, adaption, loss, and being punished. Participants were asked to answer whether each negative event occurred in the last year. Each question had a five-point scale answer which ranges from 0 (Not happen) to 1 (if occurred, but no effect) and 5 (if occurred, strong impact).

### Social support rating scale

The SSRS was a widely used 10-item questionnaire for measuring social support in the Chinese adolescent population from 3 dimensions: objective social support (3 items), subjective social support (4 items), and utilization of social support (3 items) (33, 34). Objective social support refers to material and tangible support; subjective social support refers to emotional support, which was the feelings of respect, support, and being understood. A higher score indicated more social support.

### The multidimensional scale of perceived social support scale

MSPSS was a 12-item questionnaire, which measured perceived social support in 3 dimensions: family (questions 3, 4, 8, 11), friends (questions 6, 7, 9, 12), and significant others (questions 1, 2, 5, 10) (35, 36). Each dimension had 4 questions, such as “There is a special person who is around when I am in need,” scored on a seven-point Likert scale, ranging from 1 (very strongly disagree) to 7 (very strongly agree). The sum score of 4 questions of a subscale was the total subscale score and a higher score represented higher perceived social support.

### Statistical analysis

Descriptive statistics for all the variables were first conducted using percentages for the categorical variables. For continuous variables (like age, depression, anxiety, etc.), mean, standard deviation (SD), and range were computed. Then Pearson chi-square test of association was used to test the bivariate association between NSSI and the categorical variables and one-way analysis of variance (ANOVA) was used to compare the mean value of continuous variables in adolescents who engaged in NSSI versus adolescents who did not engage in NSSI. Furtherly, the Binary logistic regression was fitted to identify risk factors for NSSI as a function of gender, age, depression, anxiety, sleep quality, etc. A series of model fitness indexes were leveraged to evaluate the general fit of the model, including the Hosmer-Lemeshow test, Omnibus Tests of model Coefficients, and Nagelkerke pseudo-R square. The Hosmer-Lemeshow test and Nagelkerke pseudo-R square, which is used frequently in risk prediction models, were employed to test for goodness of fit for logistic regression models (37, 38). The Omnibus Tests of model Coefficients were used to evaluate the statistical significance of the logistic regression model. Predictors were considered significant if the value of p was less than 0.05. Odd ratios (OR) and 95% confidence interval (CI) were recorded. All statistical analyses were conducted with R (version 4.0.2). HosmerLemeshowTest and PseudoR2 function in DescTools package (version 0.99.42) were used to conduct the Hosmer-Lemeshow test and compute the Nagelkerke pseudo-R square, respectively (39). All the data analysis R code could be found at https://github.com/wuyong0103/NSSI.

## Results

### Descriptive statistics

The demographical and clinical characteristics of the master sample were summarized in Table 1. In all, we collected 742 Chinese adolescent inpatients aged 11–19 years old. Of these participants, 51.5% (382 individuals) self-reported being engaged in NSSI. Approximately three out of four (71.3%) female adolescents had NSSI behavior. More than half of the inpatient respondents agreed with suicide (54%) and euthanasia (58.4). The mean and SD for traumatic symptoms are for depression: 14.64 (7.36), anxiety: 10.87 (5.87), stigma: 38 (9.86), insomnia: 8.81 (4.34). Childhood trauma, adolescent life events, social support, and perceived social support results were listed in Table 1.

**Table 1:** Sample characteristics (*N* = 742).

Variables	Frequency (%)	Mean	SD
Age at assessment		14.69	1.69
Engaged in NSSI			
Yes	382 (51.5)		
No	360 (48.5)		
Gender			
Male	213 (28.7)		
Female	529 (71.3)		
Depress		14.64	7.36
Anxiety		10.87	5.87
Stigma		38.00	9.86
Insomnia		8.81	4.34
Emotional abuse		10.45	4.81
Body abuse		7.49	3.74
Sex abuse		5.88	2.27
Emotional neglect		14.75	5.54
Body neglect		9.50	3.43
Attitude to suicide behavior			
Approbatory	406 (54.7)		
Neutral	231 (31.1)		
Opposed	105 (14.2)		
Attitude to suicide			
Approbatory	570 (76.8)		
Neutral	162 (21.8)		
Opposed	10 (1.3)		
Attitude to suicide dependent			
Approbatory	417 (56.2)		
Neutral	311 (42.0)		
Opposed	14 (1.9)		
Attitude to euthanasia			
Approbatory	433 (58.4)		
Neutral	248 (33.4)		
Opposed	61 (8.2)		
Interpersonal stress		9.67	6.21
Study pressure		9.84	4.88
Adaptation		9.18	4.73
Lose		7.56	5.30
be punished		8.92	6.50
Support from family		15.92	6.71
Support outside of family		32.08	12.36
Sum perceived support		47.82	17.59
Objective support		8.03	2.84
Subjective support		15.48	3.92
Utilization support		6.27	2.20
Sum social support		29.87	7.40

### Bivariate analysis

We then conducted a series of chi-square (Table 2) and one-way ANOVA (Table 3) analyses between adolescents who engaged in NSSI and who did not engage in NSSI on categorical variables and continuous variables, respectively. As shown in Table 2, we found the frequency of NSSI was significantly higher in girls (58.8%) than in boys (23.0%, *χ*^2^ = 76.43, *p* < 2.2 × 10^−16^). The attitude toward suicidal behavior was also significantly different between individuals engaged in NSSI and individuals not engaged in NSSI, with the former showing more tolerance toward suicidal behavior (74.6% NSSI individuals answered “Yes” versus 8.6% no NSSI individuals answered “Yes” to suicide behavior, χ^2^ = 249.07, *p* < 2.2 × 10^−16^). Compared to individuals engaged in NSSI, individuals without NSSI behaviors were more cautious about euthanasia (60.7% NSSI individuals answered “Yes” versus 16.4% no NSSI individuals answered “Yes” to euthanasia, *χ*^2^ = 69.02, *p* = 1.03 × 10^−15^).

**Table 2:** Bivariate association between NSSI and predictors (N = 742).

Variables	History of NSSI		Chi-square	*p*-value
	No	Yes		
Gender				
Male	164	49	76.43	< 2.20 × 10^−16^
Female	218	311		
Attitude to suicide behavior				
Approbatory	103	303	249.07	< 2.20 × 10^−16^
Neutral	183	48		
Opposed	96	9		
Attitude to suicide				
Approbatory	260	310	34.25	3.65 × 10^−8^
Neutral	114	48		
Opposed	8	2		
Attitude to suicide dependent				
Approbatory	210	207	0.58	0.75
Neutral	164	147		
Opposed	8	6		
Attitude to euthanasia				
Approbatory	170	263	69.02	1.03 × 10^−15^
Neutral	161	87		
Opposed	51	10		

**Table 3:** ANOVA result between NSSI and predictors (N = 742).

Variables	NO NSSI	NSSI	*p*-value
	Mean (SD)	Mean (SD)	
Age	15.01 (1.74)	14.36 (1.56)	1.12 × 10^−7^
Depress	10.46 (6.52)	19.09 (5.30)	<2.00 × 10^−16^
Anxiety	8.27 (5.56)	13.63 (4.86)	<2.00 × 10^−16^
Stigma	40.72 (9.19)	35.13 (9.73)	3.29 × 10^−15^
Insomnia	6.91 (3.87)	10.82 (3.89)	<2.00 × 10^−16^
Emotional abuse	9.07 (4.16)	11.91 (5.02)	<2.23 × 10^−16^
Body abuse	7.16 (3.47)	7.83 (3.99)	0.02
Sex abuse	5.73 (1.86)	6.03 (2.63)	0.08
Emotional neglect	13.45 (5.40)	16.14 (5.35)	1.73 × 10^−11^
Body neglect	8.85 (3.11)	10.19 (3.62)	7.99 × 10^−8^
Interpersonal stress	7.92 (4.29)	11.54 (7.30)	5.35 × 10^−16^
Study pressure	8.62 (4.54)	11.14 (4.91)	8.12 × 10^−13^
Adaptation	7.69 (4.37)	10.77 (4.59)	<2.00 × 10^−16^
Lose	6.79 (5.00)	8.37 (5.50)	4.51 × 10^−5^
be punished	7.65 (5.75)	10.27 (6.99)	3.26 × 10^−8^
Support from family	17.76 (6.50)	13.98 (6.39)	5.17 × 10^−15^
Support outside of family	35.42 (12.24)	28.53 (11.48)	1.05 × 10^−14^
Sum perceived support	53.10 (17.22)	42.23 (16.21)	<2.00 × 10^−16^
Objective support	8.43 (2.91)	7.61 (2.70)	7.24 × 10^−5^
Subjective support	16.20 (4.06)	14.72 (3.62)	2.11 × 10^−7^
Utilization support	6.78 (2.33)	5.73 (1.91)	4.48 × 10^−11^
Sum social support	31.49 (7.56)	28.16 (6.84)	5.28 × 10^−10^

Most of the continuous variables examined in this study were significantly associated with NSSI as shown in Table 3. In terms of age, the younger individuals seemed to be more inclined to NSSI (Mean_noNSSI_ = 15.01 versus Mean_NSSI_ = 14.36, *p* = 1.12 × 10^−7^). Consistent with other studies (40, 41), depression (Mean_noNSSI_ = 10.46 versus Mean_NSSI_ = 19.09, *p* < 2 × 10^−16^) and anxiety (Mean_noNSSI_ = 8.27 versus Mean_NSSI_ = 13.63, p < 2 × 10^−16^) were also significantly associated with NSSI in our study. Adolescents were more likely engaged in NSSI if they: had a low level of self-stigma (Mean_noNSSI_ = 40.72 versus Mean_NSSI_ = 35.13, *p* = 3.29 × 10^−15^), were disturbed by insomnia (Mean_noNSSI_ = 6.91 versus Mean_NSSI_ = 10.82, *p* < 2 × 10^−16^), were more emotionally abused (Mean_noNSSI_ = 9.07 versus Mean_NSSI_ = 11.91, *p* < 2.23 × 10^−16^), were more emotionally neglected (Mean_noNSSI_ = 13.45 versus Mean_NSSI_ = 16.14, *p* = 1.73 × 10^−11^), experienced more negative events or were more affected by negative events (Table 3, interpersonal stress, study pressure, adaption, loss and punish score were significantly higher in NSSI group compared with non-NSSI group), gained less support from society (SumSocial: Mean_noNSSI_ = 53.10 versus Mean_NSSI_ = 43.23, p < 2 × 10^−16^), had lower levels of perceived social support (SumPerceived: Mean_noNSSI_ = 31.49 versus Mean_NSSI_ = 28.16, *p* < 5.28 × 10^−10^). However, unlike some previous studies (7, 11, 42, 43), we did not observe differences in childhood sexual abuse between adolescents engaged in NSSI and adolescents not engaged in NSSI. In fact, this might be partly due to, the sexual abuse score was relatively low among all participants (Table 1, Mean = 5.88, SD = 2.27).

### Multivariate analysis

Although most of the variables in our study were significantly different between the NSSI group and the non-NSSI group, we could not confirm the net effect of each variable on NSSI. Because when we analyzed a specific variable, we could not exclude the influence of other factors on this variable. We then conducted binary Logistic regression analysis on NSSI using age, depression, anxiety, insomnia, and other variables as predictors. As results shown in Table 4, we observed gender, depression, and attitude toward suicidal behavior were the risk factors for NSSI. Females were 3.4 times more likely to be engaged in NSSI compared with their male counterparts (OR = 3.43, 95%CI = 2.08–5.74, *p* = 1.70 × 10^−6^). Each additional increase in symptoms of depression increases the odds of engaging in NSSI by 18%, net the effect of all the other predictors (OR = 1.18, 95% CI = 1.12–1.25, *p* = 2.25 × 10^−8^). Compared to adolescents with approbatory attitude to suicide behavior, adolescents, who were neutral (OR = 0.163, 95% CI = 0.10–0.27, *p* = 4.05 × 10^−12^) or opposed (OR = 0.13, 95% CI = 0.05–0.32, *p* = 1.24 × 10^−5^) to suicide behavior, were less likely being engaged in NSSI. Other factors were not significant in multivariate analysis.

**Table 4:** Binary Logistic regression analysis of NSSI (N = 742).

Variables	Estimate	SE	OR	95%CI	*p*-value
Intercept	−0.40	1.46	0.67	0.04–11.70	0.79
Gender - female	1.23	0.26	3.43	2.09–5.74	1.70 × 10^−6^
Age	−0.09	0.07	0.91	0.80–1.04	0.18
Depress	0.17	0.03	1.18	1.12–1.25	2.25 × 10^−8^
Anxiety	−0.02	0.03	0.98	0.92–1.05	0.59
Stigma	−0.01	0.01	0.99	0.97–1.02	0.57
Insomnia	0.00	0.04	1.00	0.94–1.07	0.99
Emotional abuse	−0.01	0.03	0.99	0.92–1.06	0.72
Body abuse	−0.02	0.04	0.98	0.91–1.05	0.56
Sexual abuse	0.03	0.05	1.03	0.93–1.14	0.60
Emotional neglect	−0.02	0.03	0.98	0.93–1.04	0.51
Body neglect	0.04	0.04	1.04	0.96–1.13	0.38
Attitude to suicide behavior					
Neutral	−1.81	0.26	0.16	0.10–0.27	4.05 × 10^−12^
Opposed	−2.01	0.46	0.13	0.05–0.32	1.24 × 10^−5^
Attitude to suicide					
Neutral	−0.13	0.29	0.88	0.50–1.56	0.66
Opposed	−0.67	1.26	0.51	0.04–4.90	0.60
Attitude to suicide dependent					
Neutral	−0.36	0.24	0.70	0.44–1.12	0.13
Opposed	−1.23	0.73	0.29	0.07–1.21	0.09
Attitude to euthanasia					
Neutral	0.37	0.27	1.45	0.87–2.47	0.16
Opposed	−0.17	0.55	0.85	0.28–2.44	0.76
Interpersonal stress	0.05	0.03	1.05	0.99–1.12	0.12
Study pressure	−0.04	0.03	0.96	0.91–1.02	0.17
Adaptation	0.04	0.03	1.04	0.98–1.11	0.22
Lose	−0.04	0.03	0.96	0.92–1.01	0.16
be punished	0.01	0.03	1.01	0.96–1.06	0.84
Support from family	0.07	0.07	1.08	0.96–1.28	0.30
Support outside of family	0.12	0.07	1.13	1.00–1.34	0.09
Sum perceived support	−0.12	0.07	0.89	0.75–1.00	0.09
Objective support	0.13	0.07	1.14	0.98–1.32	0.08
Subjective support	0.04	0.06	1.04	0.91–1.17	0.58
Utilization support	0.07	0.08	1.08	0.92–1.26	0.35
Sum social support	−0.07	0.05	0.94	0.84–1.05	0.22

Omnibus chi-square = 414.80 (*p* < 0.001).

Hosmer-Lemeshow G.O.F. test statistic = 9.34 (*p* = 0.32).

Nagelkerke pseudo-*R* square = 0.60.

Overall percent correctly classified = 82.6%.

The Hosmer-Lemeshow Goodness of Fit test result showed the overall fit of the model was good and the variables made a significant contribution to the model (*χ*^2^ = 4.56, *p* = 0.80). The chi-square value of the Omnibus Tests of Model Coefficients was 441.97 with a statisticaly significant *p* < 0.001. The Nagelkerker pseudo-*R* square was 0.599 which indicated that 59.9% variances of NSSI could be explained by all the predictors in the Logistic regression model. In all, 82.6% of adolescents could be correctly classified into the NSSI group and non-NSSI group according to the regression model.

## Discussion

Here, we observed a total of 51.5% prevalence of NSSI in mental clinical adolescent inpatients. This prevalence was almost equal to that of prevalence in inpatients with psychiatric disorders (40–60%) from other reports (1, 9). Evidence from genetic (44) and epidemiological (41) studies also support that NSSI had a high comorbidity with mental illness. In this study, we also found depression, anxiety, insomnia, and childhood trauma (except for sexual abuse) were significantly associated with NSSI (Table 3).

The finding that NSSI was more common in younger adolescents than in older adolescents both corroborates and contradicts previous research on NSSI. Consistent with some previous studies (23, 45, 46), age was found significantly associated with NSSI in our investigation. However, different from some of the other studies (9, 47), which reported older adolescents were more likely to engage in NSSI, we observed a totally opposite trend that younger adolescents were more likely to engage in NSSI. Muehlenkamp and Gutierrez found that by age 13, 15% of their respondents had engaged in NSSI, and 26% had engaged in NSSI as the age increased to 14, then fell to 17% when the age went to 15 (14). This trend was also found in our study with the prevalence of NSSI in ages 11–13, 13–15, 15–17, and 17–19 were 55.8, 58.5, 48.6, and 29.1%, respectively. The parabolic prevalence of NSSI with age further suggested that age was indeed a risk factor for NSSI and partially explained why age was not a significant predictor in the Logistic regression analysis. Why was the prevalence of NSSI high in ages between 12 and 15? A cross-sectional survey that used a measure of pubertal stage in individuals aged 12–15 years in schools showed that the onset of NSSI was related to the pubertal phase, especially late or completed puberty, rather than chronological age (45, 48). Another investigation also suggested that earlier-developing adolescents had a higher risk of NSSI and this increased risk attenuated as adolescents transition into adulthood (49). In addition, due to the liberalization of China’s second-child policy, parents of these adolescents at this age range were distracted by the increase in family members, so they used this behavior to attract the attention of their parents. Inherently, this pathological behavior in puberty might be related to neurodevelopmental vulnerability around this time and changes hormonally, physically, psychologically, and socially (50, 51).

Results from some investigations showed that female adolescents were more likely to be engaged in NSSI (9, 52), whereas other scholars did not find any significance between different genders (15, 53, 54). Anyhow, in our survey, we did observe more girls engaged in NSSI. This gender difference might be due to the closer relationship between puberty and the onset of NSSI in girls than in boys (48) or the different manner in which males and females responded to stress or regulate emotions (9). Moreover, some scholars believed that female patients are exposed to greater childhood maltreatment, at least in the case of sexual abuse, could explain the gender differences (55, 56). However, in our research, we failed to find a relationship between childhood sexual abuse and NSSI.

Consistent with previous studies (23, 57, 58), we found depression, anxiety, and insomnia were significantly associated with NSSI. However, in the binary Logistic regression model, anxiety and insomnia were not predictors for NSSI. This might be due to the strong correlation between anxiety, insomnia, and depression (59). In fact, we indeed observed a significant correlation between anxiety (OR = 2.16, 95% CI = 2.03–2.30, *p* < 2 × 10^−16^), insomnia (OR = 1.72, 95% CI = 1.58–1.86, *p* < 2 × 10^−16^) and depression using linear regression model in our data. In addition, evidence from genetics correlation and Mendelian randomization also suggested depression and other psychiatric disorders, like attention-deficit/hyperactivity disorder and schizophrenia were the most plausible causal risk factors for NSSI (44).

Childhood trauma and negative adolescent life events were significantly associated with NSSI (60, 61), and have also been found in this study. However, sexual abuse, which was controversial in previous studies (7, 10), was not significant in our study. We found the total childhood sexual abuse score was relatively low compared to other forms of childhood trauma. A meta-analysis of child sexual abuse in China from 27 studies suggested the total child sexual abuse for Chinese girls was lower than the international composites, which might be attributed to Chinese Confucian culture, collectivist values, or the one-child policy (62). It was also possible that Chinese people were more reserved about sex, and adolescents were generally reluctant to disclose their experiences of sexual abuse to people they did not fully trust. Regardless, other forms of childhood trauma and youth negative events were indeed related to NSSI, although they were not significant in the Logistic regression analysis. Like anxiety and insomnia, these negative events might also be correlated with depression as reported by some other studies (63, 64).

Although the attitude to suicide behavior was significantly associated with NSSI and also significant in the Logistic regression model, this was probably due to the fact that the participants in our study were inpatients. The mental and psychological state of these adolescents during the questionnaire investigation might affect their judgment on some problems. Unfortunately, we did not collect the suicide attitude data when they were discharged from the hospital. Future studies might take this factor into their consideration.

We also summarize several limitations of this study. Firstly, we only collected samples from one hospital, which might lead to sample-collection bias in our result. Since China is a multi-ethnic country with a vast territory, sampling from one place may not be representative. As more attention is paid to NSSI, future multi-center studies could solve this problem well. Secondly, also about the sample collection, we only included samples from inpatient cohorts. Samples from outpatient or community, or remote areas, were not included in this study. Since inpatients were more severe than outpatients, this might lead to a winner’s curse, in which we overestimated the effects of risk factors on NSSI. Lastly, the current study is a cross-sectional study, which only assessed the associations between depression, anxiety, insomnia, and negative life events with NSSI. Strictly, the cross-sectional study is not a type of cause-and-effect study. We need to use other methods, such as Mendelian randomization (65), animal experiments, etc., to confirm the causal relationship between these factors and NSSI.

In all, we found gender, depression, and attitude to suicidal behavior (at least during the hospital stay) were the risk factors for NSSI. Other factors like anxiety, insomnia, childhood trauma (except for sexual abuse), negative life events, and support from family and society might affect NSSI through depression. Age had a particular effect on NSSI, which might be the proxy of puberty. This reminds us that parents, schools, and society should pay more attention to the mental and physical health of children and adolescents, especially girls in puberty.

## Data availability statement

The raw data supporting the conclusions of this article will be made available by the authors, without undue reservation.

## Ethics statement

The studies involving human participants were reviewed and approved by Ethics Committee of Wuhan Mental Health Center. Written informed consent to participate in this study was provided by the participants’ legal guardian/next of kin.

## Author contributions

YW and JM designed the study. KS, AL, YL, JX, and YT recruited participants and collected the data. YW performed the statistical analysis and wrote the first draft of the manuscript. All authors had full access to all the data in the study and take responsibility for the integrity of the data and the accuracy of the data analysis. All authors contributed to the article and approved the submitted version.

## Funding

This study was supported by the Health and Family Planning Commission of Hubei Province (WJ2021F007 to JM) and the Health Commission of the Wuhan scientific research project (WX20Q02 to YW), and a critical project in the Wuhan Health and Family Planning Commission (WX17B15 to YL).

## Conflict of interest

The authors declare that the research was conducted in the absence of any commercial or financial relationships that could be construed as a potential conflict of interest.

## Publisher’s note

All claims expressed in this article are solely those of the authors and do not necessarily represent those of their affiliated organizations, or those of the publisher, the editors and the reviewers. Any product that may be evaluated in this article, or claim that may be made by its manufacturer, is not guaranteed or endorsed by the publisher.
